# Multidisciplinary Treatment Approach in a Patient with History of Nasopharyngeal Carcinoma

**DOI:** 10.1155/2014/918461

**Published:** 2014-01-09

**Authors:** Atacan Yavuz, Ömer Birkan Ağralı, Zeynep Lale Çalışkan, Dilek Türkaydın, Atilla Sertgöz, Bahar Kuru, Başak Doğan

**Affiliations:** ^1^Department of Periodontology, Faculty of Dentistry, Marmara University, Nişantaşı Kampusu, Büyükçiftlik Sok. No. 8, Nişantaşı-Şişli, 34365 Istanbul, Turkey; ^2^Department of Prosthodontics, Faculty of Dentistry, Marmara University, Nişantaşı Kampusu, Büyükçiftlik Sok. No. 8, Nişantaşı-Şişli, 34365 Istanbul, Turkey; ^3^Department of Endodontics, Faculty of Dentistry, Marmara University, Nişantaşı Kampusu, Büyükçiftlik Sok. No. 8, Nişantaşı-Şişli, 34365 Istanbul, Turkey

## Abstract

Radiotherapy in NPC patients has side effects on the dentition, which affects quality of life dramatically. This case report presents multidisciplinary dental treatment approach in a 17-year-old male patient with a history of nasopharyngeal carcinoma (NPC), which was treated with chemotherapy and radiotherapy. The adolescent patient applied to dental hospital 4 years after the radiotherapy with aesthetic and functional problems on dentition affecting psychological, social, and physical aspects of his life. The dentition of the patient demonstrated the severe destruction as a devastating side effect of radiotherapy. With a successful multidisciplinary approach, our patient's aesthetics, function, and self-confidence were obtained. Well-established procedures, which include preventative care and maintenance, can reduce the duration and expenses of the treatment and help in challenging the life-long complications of radiotherapy.

## 1. Introduction

Nasopharyngeal carcinoma (NPC) is a malign neoplasm occurring in the epithelial layer of the nasopharynx. The first symptom of the disease in the 60% of the patients is enlarged cervical lymph nodes which represents metastasis. Obstruction of the eustachian tube occurs in nearly 50% of patients, which leads to unilateral serous otitis media and hearing loss. Less frequent symptoms may be like the symptoms of temporomandibular dysfunction, which is more common for the dentist, thus complicating the problem [1]. Nasopharyngoscopy, computerized tomography and/or magnetic resonance imaging on suspicious lesions, and histological examination by biopsy are required for diagnosis [2].

All the major and minor salivary glands of the NPC patients who were treated with radiotherapy are affected by the large irradiation doses, thus leading the patients into severe and persistent xerostomia [3]. Occasionally, the most anterior portion of the floor of the mouth may stand unaffected [2]. Recovery of salivary output of patients is not significant [3].

Radiotherapy may lead to dental caries, even in the patients who had not experienced caries in lifetime [4]. The decrease of saliva secretion and alternation of its quality are the key factors of the radiation caries. Also dental tissues are affected by radiation directly, making teeth more susceptible to decalcification [4]. Other complications are oral candidiasis consequent to shift in the oral microflora, transient taste alterations, malnutrition and weight loss, and restricted movement of mandible as a sequel of fibrosis on mastication muscles [3, 5].

Osteoradionecrosis is the most devastating consequence of the radiotherapy. Irradiation damages osteocytes and microvascular structures; blood vessels thicken and marrow is replaced with connective tissue. Consequently, lack of new bone formation and decreased ability of bone healing may result in bone necrosis. Osteoradionecrosis can lead to persistent pain, bad taste, and pathologic fracture of the mandible [1].

This case report presents the multidisciplinary dental treatment of severe dental tissue loss in a 17-year-old male patient with history of NPC treated with chemotherapy and radiotherapy.

## 2. Case Report

A 17-year-old adolescent male patient applied to Marmara University, Faculty of Dentistry, with complaints in mastication, speech, and aesthetics; dryness on the oral mucosa, and thermal hypersensitivity. Moreover, he was depressed about his appearance of his teeth, thus affecting his social life.

According to his medical history, at the age of 12 the patient had noticed a solid swelling on the left side of his neck and lesion was diagnosed as NPC by incisional biopsy. Following 6-month period chemotherapy regime, 3-month of radiotherapy was accomplished. He has been under the routine control of his oncologist. During the oncologic treatment, tooth brushing was prohibited and he has not performed any oral hygiene procedures since then. Moreover, the patient was neither referred to a dentist nor informed about any oral hygiene instructions until applying to our clinic. The patient disregarded the cautions of the physicians and started smoking nearly 15 cigarettes a day and the body mass index was 16.3 kg/m^2^ regarded as underweight [6].

**Figure 1:**
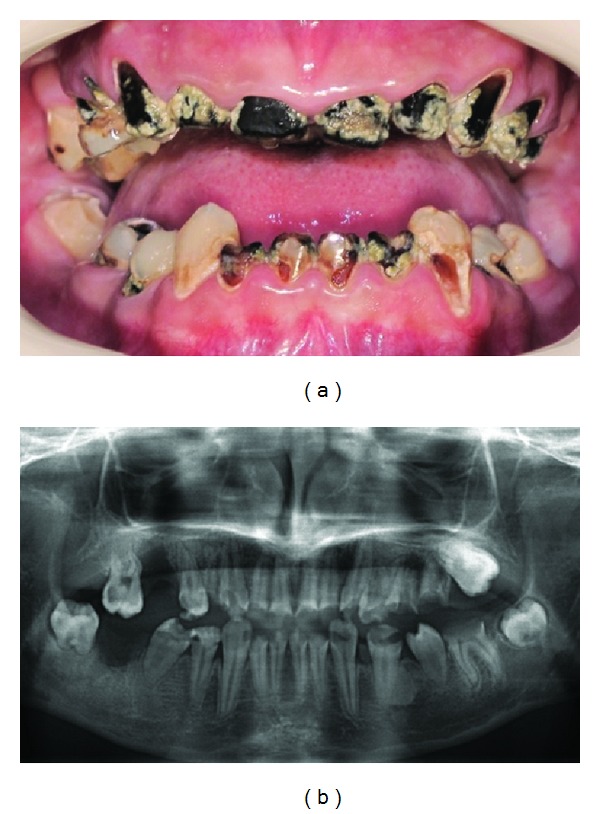
Initial intraoral (a) and radiographic (b) view of the patient.

The patient exhibited heavy plaque accumulation, xerostomia, and excessive dental caries (Figure 1(a)), as well as moderate gingival inflammation with no periodontal attachment loss. There was limited mouth opening with 25 mm interincisal distance. The radiographic examination revealed that the teeth 14, 24, 25, 26, and 36 had periapical lesions, teeth 27 and 37 were impacted, and altered teeth development was observed in teeth 37 and 47 due to oncologic treatment [7] (Figure 1(b)).

Following the oral examination, the patient was referred to his oncologist for consultation. The physician reported that he did not have any current signs or symptoms of NPC, so there was no contraindication for any dental or surgical treatments and there was no need for antibiotic prophylaxis.

A sequential multidisciplinary treatment that consisted of the extraction of the hopeless teeth, initial periodontal therapy (IPT), endodontic treatments, preprosthodontic periodontal surgery, and full mouth fixed prosthetic restorations was planned for oral health, function, and aesthetics. Written informed consent was obtained from the patient's parents after the detailed explanation of all treatments.

Due to excessive dental tissue loss and periapical lesions, the teeth 26 and 36 were extracted. Although the tooth 47 had insufficient root development and lack of bone support, the patient refused the extraction of 47 since he had no complaint about the tooth. Regarding the patient's medical history, extraction of the impacted teeth was avoided aiming not to traumatize the patient.

The patient was informed about dental plaque and its role in the gingival disease. Instructions for adequate tooth brushing and interdental cleaning were given in detail to the patient. Besides oral hygiene instructions, chewing gums with xylitol and drinking water frequently during the day were recommended to dissolve the effects of xerostomia. Moreover, the patient was encouraged to quit smoking. Microbial deposits were removed by using ultrasonic scalers (Cavitron, Dentsply Professional, USA) and hand instruments (Gracey curettes, Hu-Friedy, USA) and at the end of 6 weeks, the mean plaque score [8] decreased from 2.56 to 0.35 and gingival score [9] from 1.95 to 0.5.

**Figure 2:**
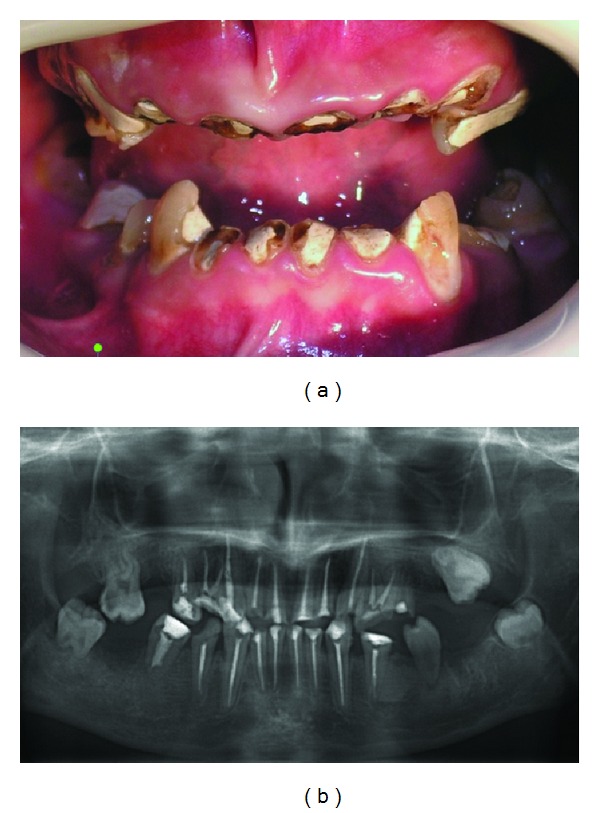
Intraoral (a) and radiographic (b) view of the patient after initial periodontal treatment and endodontic treatments.

Endodontic treatment was performed between the teeth 15–25 and 35–45 except 17 and 25 (Figure 2). The root canals of the teeth were instrumented manually under asepsis with 2.5% sodium hypochlorite irrigation and aspiration. The root canals were obturated with resin canal sealer (AH-Plus, Dentsply, Konstanz, Germany) and gutta-percha points. Lateral condensation was performed with the aid of the finger spreader. The teeth 17 and 25 were extracted since 17 had highly curved root canals and insufficient working space to perform endodontics and 25 failed to respond to endodontic treatment.

**Figure 3:**
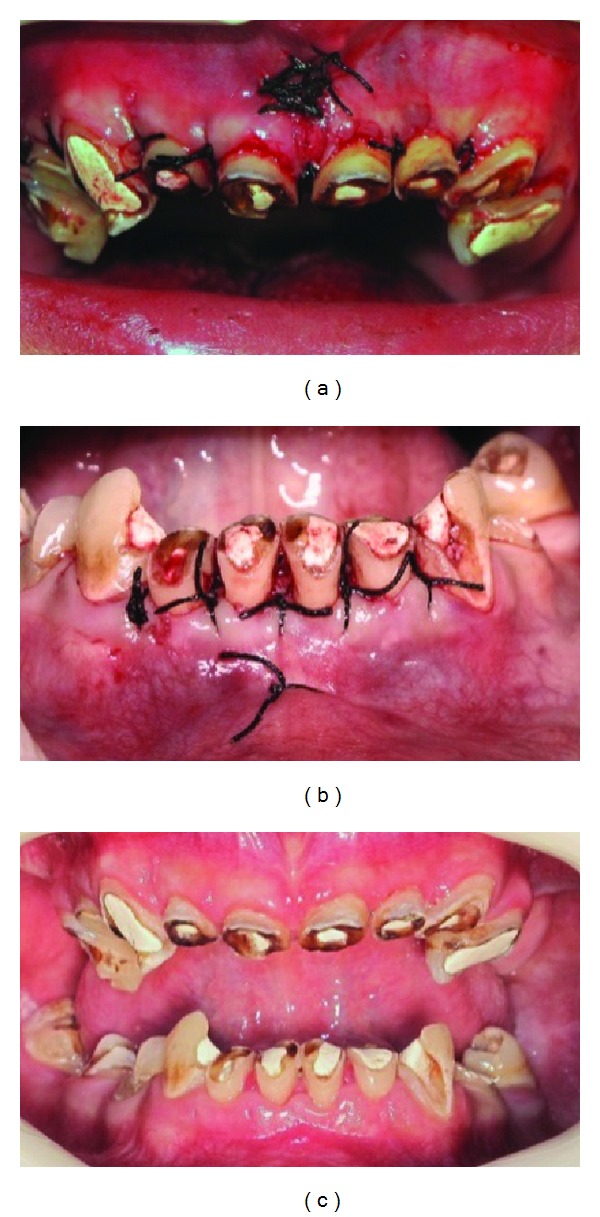
Crown lengthening procedures. Labial frenectomy and osseous recontouring on maxilla (a), apically repositioned flap technique on mandibula anterior area (b), and postoperative view at 6th week (c).

Full mouth metal ceramic veneers over postcore substructures were planned for the patient. In order to provide ferrule resistance for postcores [10] and to obtain adequate biological width for restoration margins [11], crown lengthening procedures from tooth 15 to tooth 24 and from tooth 33 to tooth 43 were performed. Labial frenectomy operation was performed prior to osseous recontouring procedure on maxilla (Figure 3(a)). Since the width of the keratinized gingival tissue was 2 mm from tooth 33 to tooth 43, apically repositioned flap technique was applied to the area to keep the keratinized tissue (Figure 3(b)) [12]. Postoperative recalls were scheduled at the 1st, 2nd, and the 6th weeks (Figure 3(c)).

**Figure 4:**
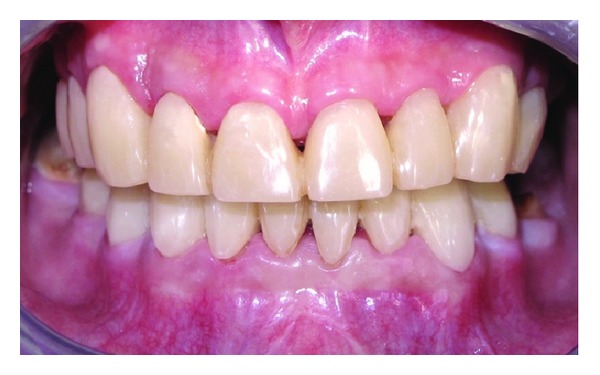
Provisional restorations with new occlusal vertical dimensions.

**Figure 5:**
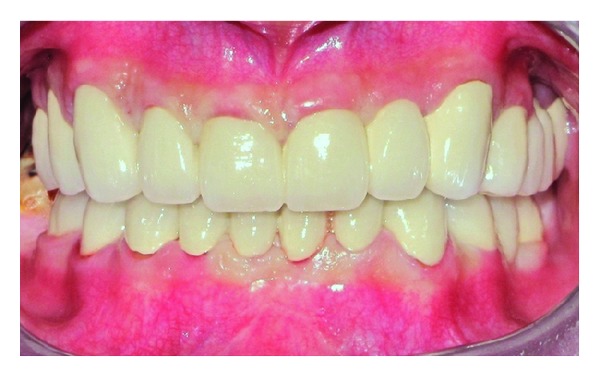
Trial of permanent prosthodontic restorations.

**Figure 6:**
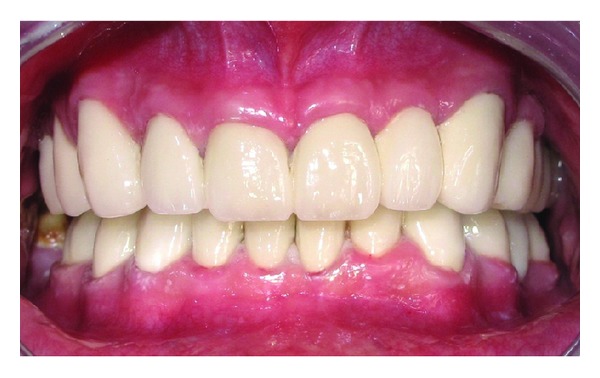
Final intraoral view after cementation of the restorations.

Prefabricated metal posts (Svenska Dentorama AB, Sweden) were placed and then composite resin cores were built (Ælite, Bisco, USA). In the same visit, exposure of the tooth 27 to the oral cavity was observed and full eruption of the tooth was decided to wait for using as an abutment. Core restorations were prepared and impression was made for provisional restorations. New occlusal vertical dimension (OVD) was established and provisional restorations were prepared in regard to new OVD (Figure 4). The provisional restorations were fitted and adjusted in the mouth. Interdental hygiene products suitable for fixed restorations (Proxybrush TePe, Sweden, Superfloss Oral-B, Procter & Gamble, USA) were instructed to the patient. Provisional restorations were maintained for 6 months, while the tooth 27 was erupting. After the final preparations were performed, impressions were taken with condensation silicone impression material (Zetaplus, Oranwash, Zhermack, Italy) using Putty-Wash technique. Trial of the ceramic veneers (Figure 5) was performed a week after the test of the metal framework. OVD and centric relations were adjusted on the permanent restorations. Restorations were maintained intraorally with provisional cementing for the orientation of the patient. At the end of the 4-week orientation period, final adjustments were made on the proximal surfaces to facilitate proper interdental brushing and flossing, and then restorations were cemented permanently using glass ionomer cement (Kavitan Cem, Spofa Dental, Czech Republic) (Figure 6).

The patient was satisfied about his new appearance and his mastication ability was significantly improved. He was controlled at the end of the first month and then maintenance visits with 3-month intervals were scheduled.

## 3. Discussion

Radiotherapy in NPC patients has side effects on the dentition, which affects the quality of life dramatically. To reduce the dental complications of the patients, dental consultation, evaluation, and treatment should be completed before radiotherapy. The oral hygiene instructions and diet survey are important. Patients should comply with professional and individual prophylactic dental practices for long-term maintenance of the oral health and challenge with chronic complications of radiotherapy. Xerostomia is the most common chronic complication of radiotherapy, which is associated with pH of oral cavity and the cariogenic activity. Saliva substitutes, systemic agents, and mechanic and taste stimulants are useful in the treatment of xerostomia [3, 13]. Chewing gums with xylitol has also mechanic stimulant effect on salivary glands. Xylitol inhibits the growth of *Streptococcus mutans* [14]. Radiotherapy patients have to prefer water rather than soft drinks with sugar and acidic ingredients [3] and must be informed about adjuvant effect of smoking on xerostomia [15].

In this case, the patient was not consulting with a dental professional before the radiotherapy. The young patient applied to our clinic in posttreatment phase with typical dental complications of radiotherapy, xerostomia and trismus resulting in caries, and loss of function and aesthetics in addition to poor oral hygiene. Moreover, the patient was a smoker. He started to drink water instead of acidic soft drinks and to chew xylitol gums in daytime. Although the patient was urged to give up smoking, he could only decrease the number of cigarettes from 15 to 5 per day.

Daily fluoride application is a lifelong requirement due to rapid progressing of initial caries to large lesions for postradiotherapy patients [2, 3]. Intake of fluoride by toothpastes is the most effective way to prevent caries due to their clinical effectiveness and social acceptability. Toothpaste with fluoride concentration of 1000 ppm or above was found to be effective in preventing caries [16]. Regular use of daily (230 ppm) or weekly (900 ppm) mouth rinses is associated with caries reduction. The patient was instructed to use toothpaste with 1400 ppm F during routine tooth brushing (Sensodyne F) and daily use of mouth rinse with 450 ppm F (Sensodyne Pronamel). Besides the personal hygiene applications, frequent visits and professional dental cleaning are important requirements for these patients [3]. Patient was recalled every three months as planned until his obligatory military recruitment.

This reported patient suffered from NPC and had radiotherapy and chemotherapy in his puberty. He applied to our clinic 4 years after the radiotherapy with the oral complications that affected aesthetics and function. Adolescents' behavior, self-confidence, and social relations with others are highly influenced by facial and dental aesthetics. By performing multidisciplinary treatment, optimal aesthetics were achieved and the adolescent patient gained his self-confidence back.

Radiotherapy patients have to be monitored before, during, and after the therapy [2]. Dental evaluation before the radiotherapy is a standard protocol for the developed countries but not a routine practice in the health systems of most developing countries like the one in Turkey. Preventative approaches before the radiotherapy could reduce the severity of the dental complications and quality of life after the treatment is as important as survival for these patients. Last but not least, role of dental professionals in the maintenance is also crucial due to life-long complications of radiotherapy.
